# Assessing and cataloguing cognitive impairment in older potential kidney transplant patients: key recommendations from the ethical and legal issues working group of ELPAT

**DOI:** 10.3389/ti.2026.16770

**Published:** 2026-07-30

**Authors:** J. A. Holland, J. S. L Partridge, R. L. Thom, I. Raburn, D. M. Summers, A. Ruck Keene, E. M. Bunnik, A. J. Cronin

**Affiliations:** 1 Department of Transplant Surgery, Renal Medicine, and Urology, Guy’s and St Thomas’ NHS Foundation Trust, London, United Kingdom; 2 School of Immunology and Microbial Sciences, Faculty of Life Sciences and Medicine, King’s College London, London, United Kingdom; 3 Department of Perioperative Medicine for Older People Undergoing Surgery, Guy’s and St Thomas’ NHS Foundation Trust, London, United Kingdom; 4 School of Population Health and Environmental Sciences, Faculty of Life Sciences and Medicine, King’s College London, London, United Kingdom; 5 Department of Renal Medicine, Royal London Hospital, Barts NHS Foundation Trust, London, United Kingdom; 6 Department of Philosophy, King’s College London, London, United Kingdom; 7 Department of Transplant Surgery, Cambridge University Hospital NHS Foundation Trust, Cambridge, United Kingdom; 8 Dickson Poon School of Law, King’s College London, London, United Kingdom; 9 39 Essex Chambers, London, United Kingdom; 10 Department of Public Health, Medical Ethics, Philosophy and History of Medicine Programme, Erasmus University Medical Centre, Rotterdam, Netherlands

**Keywords:** cognition, cognition dysfunction, equity in transplantation, ethical aspects of organ transplantation and donation, transplant

## Abstract

Cognitive impairment is common in older patients with advanced kidney disease and may influence access to and outcomes from kidney transplantation. However, its assessment and documentation within transplant evaluation remain inconsistent, with important ethical and legal implications. This position paper from the Ethical and Legal Issues Working Group of ELPAT synthesises current evidence, ethical and legal frameworks, and findings from a UK-wide national survey to propose a structured approach to the assessment and documentation of cognitive impairment in older potential kidney transplant recipients. Survey data demonstrate substantial variability in screening practices, documentation, and capacity assessment during transplant workup, contrasting with the standardised evaluation of other transplant domains. Cognitive impairment may affect decision-making capacity, perioperative risk, post-transplant self-management, and graft outcomes, raising important ethical considerations regarding autonomy, equity, and resource allocation. We recommend routine screening using validated tools, standardised documentation within clinical records and registries, use of these data to inform policy and research, prioritisation of cognitive outcomes in future studies, and targeted education and training in cognitive and capacity assessment. Embedding these measures within transplant pathways will support equitable access, informed decision-making, and improved outcomes.

## Introduction

Over the last 10 years the proportion of patients aged over 60 years on the kidney transplant waiting list has increased. It is recognised that older patients with advanced kidney disease are at risk of inferior outcomes following transplantation due to the negative influence of multimorbidity, frailty and cognitive impairment known to increase in frequency with age [1, 2]. While multimorbidity and frailty have been a focus of research in recent years, the effect of cognitive impairment on eligibility for, and clinical outcomes from, kidney transplantation has received less attention. A recent UK-wide survey showed that while many transplant units now have processes for assessing frailty and multimorbidity in older patients, cognitive impairment was less often assessed in pre-transplant work up. This is important not only because of the potential clinical consequences of cognitive impairment, but also because it raises ethical and legal issues [3].

Much of the previous discourse on this subject has started from the perspective that restricting access to transplantation without reference to robust outcome data is unjustifiable [4]. This is of course correct. However, with an ageing population and an increasing mismatch between organs available for transplantation and a growing waiting list, difficult decisions regarding access to transplantation do have to be made. It is therefore more important than ever to ensure that national bodies, guideline groups, priority setting partnerships and clinical transplant teams are informing these decisions with data driven clinical evidence and reference to justifiable ethical principles and the law.

The purpose of this paper is to provide healthcare professionals with a recommended approach to assessing and cataloguing cognitive impairment in older potential kidney transplant recipients in order to inform shared decision-making about waitlisting. We start by outlining the changing demographics of those on the transplant waiting list and explaining why this is an issue of increasing urgency. We then outline the nature of cognitive impairment as an umbrella description of numerous pathological processes and their consequences. We describe the natural history of the underlying aetiologies and the potential impact on decisional capacity and summarise the current clinical evidence (limited as it is) for its impact on transplant outcomes. We present the results of a survey showing that cognitive impairment – in the UK – is currently not routinely assessed and documented. We then consider various ethical rationales for assessing and cataloguing cognitive impairment: to promote individual health outcomes; to support patient autonomy; to safeguard equal access and to promote just allocation of scarce resource. We will argue that in order to adhere to these ethical principles, it is advisable to include screening for cognitive impairment in pre-transplant work up, and it is essential to bring cognitive impairment into the matrix of shared decision making about waitlisting and/or transplantation. Finally, we will set out what further clinical evidence might be needed to justify this stance and call for research to fill the gaps which might help to support or reject this hypothesis.

Our aim with this work is to offer evidence-based and impartial guidance to clinicians on the nuanced assessments of and discussions around cognitive impairment as a potential barrier to transplant waitlisting.

## Demographic change

The number of people aged over 65 years in the UK is expected to increase to 19 million by 2050 [5]. Chronic Kidney Disease (CKD) is more prevalent in older people. Kidney transplantation is the treatment of choice for End-Stage-Kidney-Disease (ESKD), both in terms of improved quality of life and cost-effectiveness compared to dialysis [6, 7]. It is inevitable therefore that demand for transplants in this ageing cohort will increase.

Frailty, multimorbidity (including hypertension, diabetes, and cardiovascular disease), and cognitive impairment are all seen more frequently as age increases and are much more prevalent among older individuals with CKD [8–10]. These factors have been associated with poorer outcomes following transplantation and currently are regarded as relative barriers to transplantation [11–13].

In the past, age was used as a contraindication for organ transplantation. This is changing. In the UK, NHS Blood and Transplant Kidney transplantation reports from 2014 to 2024 show an increase in the number of patients on the kidney transplant waitlist aged over 60 years from 31% to 35% and a concomitant increase in incident transplant patients aged over 60 [14].

## Cognitive impairment

As the population ages, neurocognitive disorders resulting in cognitive impairment are progressively observed [15]. The Diagnostic and Statistical Manual (DSM-5) refers to major neurocognitive disorder or dementia and minor neurocognitive disorders or mild cognitive impairment. The definitions of these conditions are shown in Table 1 [16].

**TABLE 1:** Diagnostic and Statistical Manual 5 (DSM 5) classification of major and minor neurocognitive disorders.

Characteristic	Major neurocognitive disorder (dementia)	Minor neurocognitive disorder (mild cognitive impairment)
Patient, informant or clinician-reported evidence of cognitive decline across one or more neurocognitive domains	Substantial decline	Modest decline
Objective decline in neurocognitive function on assessment	2 or more standard deviations below appropriate norms	1–2 standard deviations below appropriate norms
Impacts on activities of daily living such that no longer fully independent	Yes	No
Not attributable to delirium or other mental health disorder	Yes	Yes

The neurocognitive disorders category encompasses the group of acquired, rather than developmental, disorders in which the primary clinical deficit is in cognitive function.

Those with mild neurocognitive disorder or mild cognitive impairment (MCI) have an increased risk of developing major neurocognitive disorder or dementia in the future, especially when the domain of memory is predominantly affected [17]. However not everyone diagnosed with MCI progresses to dementia.

The overarching term ‘neurocognitive disorder’ does not differentiate between the many aetiological subtypes of dementia including Alzheimer’s disease, Lewy body dementia, and vascular neurocognitive disorder amongst others. Risk factors for vascular disease are also independent risk factors for both vascular type neurocognitive disorder and Alzheimer’s disease [18]. The relationship between vascular risk factors, cerebral blood vessel changes, medical risk factor control and the potential impact on both vascular dementia and Alzheimer’s dementia is not yet fully understood but remains a subject of ongoing research.

Despite these unknowns, it is observed that as the population ages the prevalence of neurocognitive disorders increases. It is estimated that one in 14 of those aged over 65 years have dementia in the UK. [19]. Given the coexisting vascular risk factors common in older patients with CKD it is unsurprising that studies estimate a prevalence of cognitive impairment up to 70% in the highest risk cohort CKD patients- i.e., those over the age of 60 who are receiving haemodialysis [20, 21]. Definitions of cognitive impairment vary between studies in renal populations, but older ESKD patients consistently underperform in screening assessments compared to their age-matched peers without kidney disease [22].

The identification of cognitive impairment and provision of a diagnosis in a high-risk population with CKD being considered for kidney transplantation is therefore relevant for several reasons. First, a diagnosis of cognitive impairment enables treatments to delay progression of dementia and proactively manage behavioural symptoms in addition to access to multidisciplinary support for patients and their carers. Second, cognitive impairment has a potential impact on mental capacity and requires appraisal prior to the process of informed consent. Third, cognisant of the close link between dementia and delirium, a proactive diagnosis allows clinicians to employ preventative strategies to reduce incidence and/or severity of delirium during the postoperative period or in subsequent hospital admissions. Fourth, acknowledging the life limiting prognosis conferred by dementia, is key to informing realistic choice regarding kidney transplant and other treatments to facilitate meaningful shared decision making and if appropriate to prompt advanced care planning and treatment escalation planning [23]. Fifth, in those who receive kidney transplants, cognitive impairment may be associated with challenges in medication adherence and self-management, which could contribute to poorer transplant outcomes in some recipients. Available evidence suggests that outcomes may be less favourable in certain cognitively impaired populations, although the evidence base remains limited and largely observational. These considerations may support the provision of enhanced monitoring and support following transplantation [24, 25].

Importantly, cognitive impairment in advanced kidney disease should not be considered solely through the lens of the potential risk conferred upon transplantation. CKD and dialysis themselves are associated with cognitive decline, while several studies have demonstrated improvement in selected cognitive domains following transplantation [13, 20, 21]. Consequently, for some patients, transplantation may represent not only a treatment for kidney failure but also an opportunity to stabilise or improve aspects of cognitive function.

## Assessment of neurocognitive disorders

As illustrated in Table 1 the diagnosis of either major or minor neurocognitive disorder relies on the combination of self or observer reported cognitive decline and objectively impaired cognition evaluated using neurocognitive assessment. Different methods of cognitive assessment exist, ranging from full neurocognitive assessment batteries which take several hours to complete, to brief cognitive assessment tools. The benefits and drawbacks of these assessment tools depend on the environment in which they are being used. For example, a full neurocognitive assessment battery may be appropriate in a memory clinic where each patient is allocated a 3-h appointment time and a neuropsychologist is present to undertake and interpret the evaluation. This tool however would not be suited to a transplant clinic in which patients may need more rapid cognitive assessment prior to consideration of a surgical procedure. Whilst in this scenario a briefer cognitive assessment tool would be more suitable, consideration should also be given to the properties of the brief assessment tool, including how effectively it examines the type of cognitive impairment that is anticipated, whether it is appropriate to the educational level of the population and whether it adequately evaluates sufficient cognitive domains. For example, in a CKD population in whom vascular disease frequently coexists and therefore executive dysfunction attributed to vascular neurocognitive disorder is likely to be frequently encountered, choosing the Montreal Cognitive Assessment (MoCA) (Appendix 1) in place of the Folstein Mini Mental State Examination (MMSE) as a brief assessment tool, may be more appropriate as the MoCA better evaluates executive functioning. Where concerns exist that educational attainment level may influence assessment of cognition, the Rowland Universal Dementia Assessment Scale (RUDAS) offers a brief, standardised cognitive screening tool that assesses multiple domains of cognition, including memory, visuospatial orientation, praxis, language, and executive function. It takes approximately 10 min to administer and is specifically designed to be less influenced by language, education level, and cultural background than other tools like the Mini Mental State Examination (MMSE). Finally, contrary to other publications, proactive cognitive assessment should be undertaken prior to consideration of kidney transplantation as opposed to after surgery for the reasons stated above [1].

It is important to be clear here that cognitive impairment does not equal lack of decision-making capacity. Nevertheless, it is helpful to frame cognitive impairment as something which may lead to specific legal obligations in the way we assess and treat people where it does lead to incapacity. We acknowledge that a significant proportion of those with cognitive impairment may have impaired decision-making capacity. But at the outset given that capacity assessments are time and decision specific, the only way in which it would affect the transplant assessment at the outset is the decision about whether or not to embark upon the transplant assessment process which may, or may not, result in an individual being listed waiting for a transplant.

## Assessment and documentation of cognitive impairment is lacking

A recent UK-wide survey of all 72 renal units and 23 adult transplant centres provides important context for current practice around cognitive impairment and capacity assessment in older potential kidney transplant recipients. While most centres reported routine assessment of frailty (33/53 renal units) and multimorbidity, cognition was far less consistently evaluated. Twenty-eight of fifty-three renal units reported that cognition was “rarely” or “never” assessed in patients over 60 with advanced kidney disease, and seven of nineteen transplant centres reported rarely assessing cognition in older transplant candidates. Where cognitive screening was undertaken, tools varied: 58% of renal units used the MMSE and 32% used the MoCA, while others relied on clinical judgement or *ad hoc* approaches [3].

Concerns extended beyond screening to documentation and governance. A majority of respondents did not believe their centre had a robust method of assessing (renal units 28/54; transplant centres 10/18) or documenting (renal units 35/54; transplant centres 10/18) mental capacity in older potential transplant recipients [3]. These findings contrast sharply with the structured, protocolised and highly resourced assessment pathways for immunological risk, cardiovascular fitness and virology that characterise contemporary transplant work-up. It is noteworthy that the neither the UK transplant registry, the Dutch Organ Transplant Registry or Eurotransplant currently catalogue cognitive impairment assessments of any potential transplant recipients.

Only six UK units currently provide dedicated comprehensive geriatric assessment (CGA) services for older kidney patients. Reported barriers included lack of funding (45/51), lack of trained staff (44/51) and time constraints (36/51) [3].

Where implemented, CGA involves structured multidomain assessment incorporating cognition, mental capacity, frailty, multimorbidity and psychosocial context within a reproducible clinical framework [35]. In this setting, CGA has potential utility not only in systematically identifying cognitive impairment and clarifying decision-making capacity, but also in contextualising these findings alongside frailty and multimorbidity, thereby informing optimisation, shared decision-making and equitable listing decisions. Such an approach offers an evidence-based and proportionate response to cognitive vulnerability, aligning its assessment with the sophistication and governance already embedded in other domains of transplant evaluation.

## Ethical issues

There are various ethical rationales for assessing and cataloguing cognitive impairment in older potential kidney transplant recipients with CKD stage 5 either pre- or on dialysis.

First, it may serve to improve health outcomes of individual patients, by allowing patients, caregivers and healthcare professionals to mitigate medical risks associated with cognitive impairment. The identification of cognitive impairment or dementia allows for timely referral to specialist services including access to symptomatic medications, medications to delay progression of disease and support services (for both the patient and carers) which have benefits in minimising crisis admissions and carer stress. Identification of cognitive impairment or dementia also allows for employment of appropriate care to mitigate risks related to cognitive impairment or dementia if particular treatments are undertaken e.g., using a delirium bundle to reduce the risk of postoperative delirium following renal transplantation in the context of mild cognitive impairment. Following renal transplantation, supporting strategies might be offered to recipients with cognitive impairments to mitigate the risks listed in Table 2. Medical non-adherence, for instance, may be countered using e.g., close involvement of healthcare personnel (e.g., pharmacist, nurse) in drug distribution, the use of electronic reminders (e.g., alarmed drug container), drug management plans, and therapy simplification [36]. It should be noted that these have not proven effective on the long term. The impact of cognitive impairment on transplant-related outcomes is likely to be modified by the presence of reliable caregivers and social support. Such support may facilitate medication adherence, attendance at follow-up appointments, and engagement with post-transplant care, and should therefore be considered alongside cognitive assessment when evaluating transplant candidacy.

**TABLE 2:** Types of cognitive impairment and impact on transplantation.

Type/Pattern of cognitive impairment	Definition	Pre-listing and evaluation	Capacity & consent	Perioperative risk	Post-transplant self-management	Graft & survival outcomes	Ethical considerations	Potential mitigation*
Mild cognitive impairment (MCI) [24, 25]	Objective cognitive decline in ≥1 domain (e.g., memory, executive function) with preserved independence in basic activities of daily living	Associated with lower likelihood of listing and longer time to listing [13, 26]	Capacity to consent often preserved; may require enhanced communication [3]	Possible increased delirium vulnerability [7]	Increased risk of medication mismanagement without support [27, 28]	Some domains improve post-KT [29, 30]; long-term deficits may persis [9]	Should not constitute automatic exclusion; requires supported decision-making	Routine screening (MoCA/RUDAS); caregiver involvement; adherence support
Executive dysfunction (vascular pattern) [27, 30, 31]	Predominant impairment in planning, attention, processing speed, and cognitive flexibility, often associated with small-vessel cerebrovascular disease	Difficulty navigating complex evaluation pathways [26]	Often intact capacity to consent but impaired planning/reasoning [31]	Increased susceptibility to perioperative delirium [29]	Impaired adherence and complex regimen management [27]	Indirect impact on graft outcomes via non-adherence [24, 25, 29]	Executive dysfunction ≠ incapacity; risk of inequitable exclusion	Executive function screening; simplified regimens; structured follow-up
Moderate global cognitive impairment [24, 26, 29]	Impairment across multiple cognitive domains with emerging functional dependence in instrumental activities of daily living	Reduced likelihood of listing and prolonged evaluation [13, 26]	Capacity to consent may be impaired; formal assessment required	High complication and delirium risk [29]	High dependence for medication/appointments	Associated with graft loss and mortality in some cohorts [29]	Proportionality and benefit–burden analysis critical	Multidisciplinary review; formal capacity assessment; documented support plan
Established dementia (mild–moderate) [29, 32]	Progressive neurocognitive disorder causing cognitive decline sufficient to interfere with independence in daily activities	Often excluded in practice [32]	Frequently impaired capacity to consent	High perioperative and delirium risk [33]	Dependent on caregiver support	Increased graft loss (HR ∼1.5) and mortality (HR ∼2.4) post-KT [32]	Raises questions of long-term benefit horizon and justice	Individualised review; advance care planning; structured caregiver engagement
Advanced dementia [23]	Severe neurocognitive disorder with profound cognitive impairment and complete dependence in daily activities	Rarely listed	Capacity to consent to complex procedures very likely to be absent	Very high perioperative risk	Fully dependent	Limited survival horizon [23]	Often considered reasonable contraindication based on proportionality	Focus on conservative management and goals-of-care discussions
Delirium vulnerability/Fluctuating cognition [23, 32, 33]	Acute or fluctuating disturbance in attention and awareness, often superimposed on underlying cognitive impairment	May be under-recognised without screening	Fluctuating capacity; requires repeated assessment	High incidence of post-KT delirium [29]; delirium associated with later dementia [23, 34]	May accelerate cognitive decline	Associated with adverse outcomes	Reversibility must be distinguished from progressive impairment	Delirium prevention protocols; perioperative geriatric input

* Mitigation in this context refers to interventions or strategies that may reduce the impact of cognitive impairment on transplant-related processes and outcomes.

Second, routine assessment of cognitive impairment in older potential kidney transplant recipients may help promote patient autonomy. Clinicians need to inform patients and their proxies about additional medical risks, about the prognosis from a life limiting condition such as dementia and potentially more limited benefits of organ transplantation in the context of cognitive impairments, and the need, availability and limitations of supporting strategies in the post-transplant setting to optimize medication adherence and therewith, graft functioning. Given the additional risks, possibly limited life expectancy and more limited effects on quality of life associated with certain types of cognitive impairment, the identification of cognitive impairment may tip the balance of risks and benefits associated with organ transplantation for older patients. Adequate information about the prognosis of the condition underlying cognitive impairment may help older patients and proxies decide whether or not organ transplantation is in line with patient interests, values and preferences. Also, routine assessment of cognitive impairment may help clinicians anticipate challenges related to information provision, shared decision-making and informed consent. Clinicians may need to adapt the decision-making process with patients and proxies, e.g., simplifying the language, making more time, in support of patient autonomy, so as to optimally engage patients with cognitive impairment in shared decision-making about waitlisting.

A third ethical rationale for routinely assessing cognitive impairment is to promote equitable access to transplantation, primarily by monitoring the inclusion of patients with cognitive impairment in transplantation and taking action if deemed appropriate. From a perspective of formal justice requirements, it is important to note that there is potentially currently great inequity in accessing the transplant waitlist. Cognitive impairment is recognised by transplant physicians as important [37]. However, evidence suggests different approaches are being used by different Professionals. There is a lack of consensus among transplant clinicians on which approach should be used [38], agreement that it is important that any assessment of cognitive impairment is transparent and robust. Formal screening using a validated tool is therefore supported. Further, from a more substantive perspective, it should be stressed that cognitive impairment is not meant to serve as an absolute exclusion criterion for waitlisting older patients for renal transplantation. It is important to note that some evidence indicates that transplantation might have positive effects on cognitive function in some patients [39]. If we discriminate cognitive impairment vs. non cognitive impairment, e.g., if we refuse to waitlist patients with cognitive impairment or refuse to allocate organs to patients with cognitive impairment, we should be doing this on the basis of medical criteria or patient preferences only.

It is essential to also recognise that cognitive screening itself may introduce inequities if applied uncritically. Performance on cognitive assessment tools may be influenced by educational attainment, language, culture and socioeconomic factors [40]. Consequently, screening results should be interpreted within the wider clinical and social context and should prompt further assessment rather than act as a stand-alone determinant of transplant eligibility. Use of culturally appropriate tools and appropriate assessor training are therefore essential.

So, routine assessment of cognitive impairment might help prevent clinicians from erring on both sides: it might help prevent some patients from being inappropriately listed (e.g., because of insufficient understanding of there being a less favourable balance of benefits and risks), and it might help prevent other patients from being denied access based on certain types of cognitive impairments, the adverse consequences of which might have (easily) been addressed using appropriate care and support mechanisms.

The three rationales discussed above are directly linked with the interests of patients with cognitive impairment and seem ethically justifiable. Another possible rationale to assess cognitive impairment is related to the utilitarian imperative to make efficient use of donor organs–as scarce resources. This rationale implies that cognitive impairment, once identified, should serve as (relative) exclusion criteria for waitlisting, on the grounds that scarce organs had better be allocated to recipients who may have fuller capacities to experience the benefits of transplantation. This consideration may be more prominent in deceased donation than in living donation, as the problem of scarcity is less absolute in living donation. From an ethical perspective, this consideration is more problematic and it may conflict with the abovementioned legal rights of patients and in particular of persons with limited decision-making capacity. It is not entirely clear to what extent considerations of utility ((quality of) life years to be gained) may pay a role in decisions regarding the waitlisting of cognitively impaired patients. As said, in some cases, there may be valid medical reasons (e.g., in case of (very) limited life expectancy, or in case transplantation would be futile) or ethical reasons (e.g., in case patients or their proxies prefer not to proceed with transplantation, or decide that the risks do not outweigh the benefits) to exclude patients with cognitive impairments. However, decisions to exclude patients with cognitive impairments from being waitlisted should not be taken arbitrarily and without valid medical or ethical reasons.

In sum, by routinely assessing cognitive impairment, clinicians can support health outcomes, patient autonomy and equitable access for older patients being considered for waitlisting for renal transplantation.

## Legal issues

Here, as so often, ethical and legal considerations march hand-in-hand. Pulling together the threads from above, there are two key legal issues that are in play in this context.

The first is an understanding of the distinction between cognitive impairment and decision-making capacity. The former is a clinical phenomenon which (depending on its cause) may serve as a contra-indicator to the person being put forward for transplantation. The latter is a socio-legal construct which requires workarounds for the person’s (legal) inability to make relevant decisions. By way of example, these workarounds in England & Wales are contained in the Mental Capacity Act 2005 [41].

The second is the potential for discrimination to arise where those with cognitive impairments are not put forward for transplant, especially where they are not put forward on the basis that they are considered to have impaired decision-making capacity. Discrimination arises where there is unjustified differential treatment: put shortly, just because a person cannot make relevant decisions is not, in and of itself, a justification for not putting them forward for transplant. Again, by way of example, in England & Wales, the obligations to secure against discrimination would arise by way of the Equality Act 2010 [42], and the Human Rights Act 1998 [43], importing into English law the non-discrimination requirements under Article 14 read together with Articles 2 and 8 of the European Convention on Human Rights [44].

## Conclusion and key recommendations: Towards routine assessment of cognitive function

We have noted that evidence on the medical risks and benefits of renal transplantation in older recipients with various types of cognitive impairment is currently lacking and we have set out ethical rationales and legal considerations in support of routine assessment of cognitive function as part of the transplant work-up assessment process. By systematically assessing cognitive function in transplant work-up, and making these data available for research purposes, studies could show how cognitive impairments affect transplant outcomes in specific patient groups. This could lead to the development of better screening tools or protocols to identify patients at risk of difficulties with decision-making and informed consent, and, more importantly, patients at risk of worse transplant outcomes. To address these risks, tailored interventions could be developed and offered at the time of, and after, waitlisting and transplantation. These interventions may include cognitive rehabilitation (i.e., strengthening cognitive skills or compensating for deficits), medication adjustments, social support, cognitive or mental health interventions, and patient and caregiver support.

Adequate documentation of cognitive impairment in patients who are being considered for kidney transplant and/or transplanted is an important first step in gathering knowledge about the consequences of cognitive impairment. It would allow for formal studies on the impact of cognitive impairment on transplant outcomes, including post-transplant medication adherence, complications, long-term graft survival, and (the quality of) decision-making and informed consent. A better evidence base would help us understand to what extent various types of cognitive impairment are associated with sub-optimal transplant outcomes, why this is so, and whether and how this can be addressed. In the consultation room, it could facilitate shared decision-making with individual patients and their families about waitlisting and transplantation.

Finally, we have set out five key recommendations to support assessment of cognitive function and shared decision making prior to transplant waitlist registration. We envisage that incorporating these recommendations into routine practice will serve to optimise access to and outcomes from transplantation.

## Recommendations to inform shared decision making prior to transplant wait list registration

### Recommendation 1: Screening assessment of cognitive function

All older potential kidney transplant recipients with CKD stage 5 either pre-dialysis or on dialysis should undergo a screening assessment of cognitive function including:

1. Completion of a brief cognitive assessment tool, Montreal Cognitive Assessment tool (MoCA) or Rowland Universal Dementia Assessment Scale (RUDAS); and
2. A clinical/collateral history of cognitive impairment or cognitive change.

If this screening assessment identifies possible cognitive impairment, timely referral to specialist services to confirm/refute diagnosis and initiate treatment and follow up is recommended. In addition, more detailed consideration of the patient’s capacity to make relevant decisions will be required including, where necessary, deciding what support can be given to enable the patient to make their own decisions.

### Recommendation 2: Documentation of cognitive function

All information about cognitive function should be documented clearly, in a standardized manner, such that it is reproducible across clinical and research settings. Ideally this should be available from

1. The patient’s electronic patient record
2. The national transplant data base, for example, The UK Transplant Registry (UKTR), the Dutch Organ Transplantation Registration or Eurotransplant

### Recommendation 3: Guidance for policy, practice and research

All data that is catalogued about cognitive function on the national or international transplant databases, for example, The UK Transplant Registry, should be available for use to inform transplant wait listing protocols, national or international organ allocation policy, and support ongoing and future research.

### Recommendation 4: Research and innovation

Clinical and academic researchers, funders and priority setting partnerships should be encouraged to include cognitive measures in outcome studies in older patients with CKD stage 5 either pre-dialysis or on dialysis. Inclusion of cognitive measures/diagnosis in national datasets will provide useful resource for the wider transplant research and innovation community.

### Recommendation 5: Education and training in cognitive function and decision-making capacity assessment

All clinicians involved in the assessment of older potential transplant recipients should undergo education and training in assessment of cognitive function and decision-making capacity. Funding should support training module development and embedding training programmes.
